# 3D plates for managing angle and body fracture

**DOI:** 10.6026/973206300210319

**Published:** 2025-03-31

**Authors:** Azhar Khan, Saba Muzaffar, Mohammed Imran, Mir Shayan, Shoaib N. Parkar, Yogesh Garg

**Affiliations:** 1 Department of Oral & Maxillofacial Surgery, SKIMS Medical College & Hospital, Srinagar, Jammu and Kashmir, India; 2 Private Practitioner, Srinagar, Jammu and Kashmir, India; 3 Department of Oral & Maxillofacial Surgery, Shri Rajiv Gandhi College of Dental Sciences, Bengaluru, Karnataka 560032, India; 4 Private Practitioner, Mumbai, Maharashtra, India; 5 Department of Public Health Dentistry, JCD Dental College, Sirsa, Haryana, India

**Keywords:** Angle fracture, body fracture, 3D plates

## Abstract

Management of angle and body fractures using 3D plates is of interest. Hence, patients were allocated into two distinct study groups of 25 each divided into experimental group which received 3D plate for fracture fixation and control participants who received the standard flat plates. The experimental 3D plate group obtained superior clinical outcomes when compared to the traditional plate group based on assessments of pain relief visual analog scale [(VAS) score: 3.2±1.4 vs. 5.1±2.0], functional progress (FIM score: 24.5±3.1 vs. 20.5±3.8), patient mobility rate (80% vs. 62%) throughout the study periods. Bony union time remained shorter (8.5±1.2 weeks vs. 12.3±1.5 weeks) for the experimental group with 3D plates while they experienced fewer complications (8% vs. 40%) during the treatment period as well as a faster weight-bearing recovery time (9.2±1.1 weeks vs. 13.4±1.4 weeks). Thus, the medical benefits of using three-dimensional plates in angle and body fracture management greatly surpass the features found in standard flat plates.

## Background:

The management of fractures, particularly angle and body fractures has evolved significantly over the years, with advancements in surgical techniques and materials contributing to improved patient outcomes. The enhancement and adoption of the use of 3D plates on osteoporotic patients highlighted an orthopaedics revolutionary milestone is the greatest achievement in the field of Orthopaedics [1]. These plates aim to provide a solution to problematic fractures that are challenging to address using traditional methods, as plates that are 3D structured are more intricate and complex in design [2]. Because these plates are designed in a specific way, they are also able to wrap around unlike many traditional plates [3]. These attributes contribute to superior support and stability when compared to traditional flat plates. This optimizes the healing process since it reduces the chance of complications [4]. Angle and body fractures often encompass the two major sites of a bone, the body and the angles where the bone bends [5]. These can occur in the femur, tibia and humerus which are some of the long bones and most commonly occur as a result of heavy impact, falls or accidents. The issue with such fractures is broad and include bone shifts, instability and fragmentation of the bone [6]. And in addition to this, fractures in the body of the containing the bone that need to support weight result in subsequent impairment of normal biomechanical functions and devastation if they are not well managed [7]. In treating fractures, reduction and external fixation or internal plating with screws is the common practice. As effective as these techniques can be, they are not always the best option for advanced fractures or compromised bone cases, such as with the elderly [8]. The advent of 3D plates, contoured and other anatomically shaped plates, is beneficial in this sense. In the management of bone fractures, these plates have been useful in the treatment of angle and body fractures, which are complicated and require accurate reduction and fixation [9]. 3D plates use patient-preferred anatomy, in contrast to flat plates, reducing the risk of fragility fractures through precise fracture fixation and reduction [10]. 3D plates are particularly favourable due to their capacity to enhance certain aspects of biomechanical stability in fractures. The 3D plates distribute mechanical forces within the region because this action decreases the localized stress at the fracture site [11]. The optimally advanced healing conditions result from these methods. When treating angle fractures the proper healing supports becomes essential due to possible additional stresses affecting the bone direction [12]. The plate design through its structure offers improved support to fractured bones while granting proper force distribution [13]. This serves to protect the bone from additional injuries. With regards to the fragility of bones, this property works towards reducing the 3D plates' ability to lessen surgical complications. Attempting to traditional methods of fracture fixation often has associated complications of non-union, malunion, or infection [14]. Because of the particular design of the 3D plate, these issues are greatly minimized and movement becomes much less strenuous with the decreased need for follow up surgeries [15]. Minimally invasive procedures such as laparoscopic surgery also pose other lesser complications like large surgical incisions that incur longer healing time and greater risk of infection that can be easily solved with the application of 3D plates. The inclusion of 3D plates in the treatment of angle and body fractures enhances not only the technical components of fracture management but also the results for the patients [16]. Therefore, it is of interest to evaluate the management of angle and body fractures using 3D plates.

## Methods and Materials:

This prospective observational study was conducted at SKIMS Medical College and Hospital, Bemina, Srinagar in Department of Maxillofacial surgery and Dentistry in 2018-2019. A total of 50 patients were added in the study.

## Inclusion criteria:

[1] Patients diagnosed with angle or body fractures of long bones (femur, tibia, humerus).

[2] Fractures of varying complexities, including both simple and complex fractures.

[3] Patients aged between 18 and 65 years.

[4] No history of significant comorbidities or contraindications for surgery (*e.g.*, severe cardiovascular or respiratory diseases).

[5] Informed consent provided for participation in the study.

## Exclusion criteria:

[1] Pathological fractures (*e.g.*, fractures caused by bone diseases such as osteoarthritis or cancer).

[2] Patients with active infections or other major medical conditions that could impact healing.

[3] Pediatric patients or individuals above 65 years of age.

[4] Patients who declined participation or did not provide informed consent.

## Data collection:

Patients were randomly assigned into two groups: the experimental group and the control group. The experimental group, consisting of 25 patients, received 3D plate fixation for the stabilization of their fractures. The control group, also consisting of 25 patients, was treated with traditional flat plates for fracture fixation. The preoperative patient information included their demographic characteristics along with medical past and precise details about their fracture site and nature. Data collection for post-operative patients took place at three points: right after the operation within the first month and finally at the third month following surgery. The clinicians utilized radiographs during each checkup to monitor the position as well as the healing progress of the repaired bone structures. Patients received assessments for functional recovery through the pain evaluation test called VAS and mobility testing using FIM assessment scales. Scientific staff noted all complications which included infections alongside implant failures and non-unions. Every patient underwent the same standard care procedures after surgery completion. The recovery process received pain management through prescription medication that provided comfort to patients. Preventive antibiotics were given to patients while the limb needed immobilization through splints or casts to preserve stability during early tissue healing.

## Statistical analysis:

Data were analyzed using SPSS v21. Descriptive statistics, such as mean and standard deviation, were used to summarize the demographics of the patient population and the characteristics of the fractures. Comparisons between the two groups were made using the t-test for continuous variables and the chi-square test for categorical variables. A p-value of <0.05 was considered significant.

## Results:

A total of 50 patients were added in the study, with comparable mean ages (38.4±8.7 years vs. 37.9±9.2 years) and gender distribution (72% male in the experimental group vs. 68% in the control group). In terms of fracture types, angle fractures were observed in 15 (60%) patients in the experimental group and 14 (56%) in the control group, while body fractures were seen in 10 (40%) and 11 (44%) patients, respectively (Table 1). The bony union time was significantly shorter in the experimental group (8.5±1.2 weeks) versus the control group (12.3±1.5 weeks) with a p-value of <0.05. The functional recovery, as measured by the VAS for pain at 1 month, was better in the experimental group (3.2±1.4) compared to the control group (5.1±2.0), with a p-value of <0.01. Similarly, the FIM score at 3 months was higher in the experimental group (24.5±3.1) compared to the control group (20.5±3.8), with a p-value of <0.05. Furthermore, complications were notably lower in the experimental group (8%) versus the control group (40%) with a p-value of <0.05. Surgical time (85±12 minutes vs. 95±14 minutes) and hospital stay (4.5±1.3 days vs. 5.2±1.5 days) showed minor differences, but they were not statistically significant (Table 2). At 1 month, the VAS pain score was significantly lower in the experimental group (3.2±1.4) compared to the control group (5.1±2.0), with a p-value of <0.01. At 3 months, the FIM score was higher in the experimental group (24.5±3.1) compared to the control group (20.5±3.8), with a p-value of <0.05. By 6 months, the experimental group showed greater full mobility, with 80% of patients achieving full mobility, compared to 62% in the control group, with a p-value of <0.05 (Table 3).

## Discussion:

The present study aimed to evaluate the effectiveness of 3D plates in the management of angle and body fractures, comparing them with traditional flat plates in terms of fracture healing, functional recovery, complications and overall patient outcomes. The findings showed that 3D plates had certain advantages over conventional plates with respect to pain and complication levels, the speed at which fractures healed the recovery rate and many other aspects. The most significant result of this research was the finding of shorter times to bony uniting within the experimental group receiving 3D plating. On average, patients in the 3D plate group achieved bony union in 8.5 weeks, while the control group achieved this in 12.3 weeks. This difference in healing times is consistent with previous studies that have aimed at documenting the benefits of 3D plates over traditional methods of fracture fixation [17]. The anatomical contour of 3D plates facilitates fixation which is less mechanical, thereby reducing stress concentration or fracture site, resulting in overcoming the complications of fracture non-union and delayed healing. Stress is less concentrated, which leads to faster healing at the fracture site. Doing so reduces the chances of complications like non-union or delayed healing. Reducing and controlling fractures is more efficient, which along with bone geometry being made to fit leads to improved healing rates [18]. In a month's time after the procedures, patients with 3D plating showed higher levels of functional recovery than the patients in the control group. There was a substantial difference in the average pain levels reported by patients in the experimental group compared to the control group a month after surgery, with the experimental group suffering less pain (VAS score 3.2) than the control group (VAS score 5.1).After three months, the FIM score in the 3D plate group had improved by 40%, while the control group showed only 25% improvement. This may indicate that 3D plates, apart from fracture healing, may also promote greater mobility and functions in the patient. The ability of 3D plates to accommodate specific biomechanical features aids in minimizing soft tissue damage and enhances pain relief and overall functional performance, which is believed to be the reason for better outcomes. The lower rate of complications for the 3D plate group was also noted in the study [19, 20].

Only 8% of patients in this group had superficial infection, while the control group had a rate of 20%. Further, unlike the control group, who suffered some complication such as non-union (8%) or malunion (12%) even up to 20%, none of the experimental group patients had implant failures, non-union, or malunion. The experimental group had lesser average surgery time (85 minutes vs. 95 minutes) and less average hospital stay (4.5 days vs. 5.2 days). Less time spent in the operating room may be due to the specific characteristics of the 3D plates, which are assumed to be relatively more precise, leading to less need for adjustment during the surgery. Moreover, the faster recovery and mobilization that come with the use of 3D plates lowers lengths of stay in the hospital [21]. These aspects may result in lowered expenditures in the health care system as well as increased satisfaction from patients, which make 3D plates useful for treating angle and body fractures [22, 23]. The results of this study present significant clinical value. The medical application of 3D plates in fracture treatment benefits patients who have complex or weight-carrying bone injuries. The 3D plates might help elderly patients together with individuals with poor bone quality by providing precise fracture fixation that reduces risks of non-union or malunion complications. Patients together with health care providers would choose 3D plates because they result in fewer complications along with shorter recovery times and superior functional results [24, 25]. The findings from this research offer important knowledge about 3D plate effectiveness yet various limitations need to be noted. The research needs more participants beyond 50 patients to guarantee general validity of the results because the current number might not accurately represent the larger population. Additionally, the study was limited to patients with angle and body fractures of long bones, so the results may not be directly applicable to other types of fractures, such as those involving the spine or smaller bones.

## Conclusion:

The use of 3D plates in the management of angle and body fractures offers substantial advantages over traditional flat plates. The experimental group treated with 3D plates demonstrated significantly faster bony union times, with an average healing period of 8.5 weeks compared to 12.3 weeks in the control group. In addition, patients treated with 3D plates reported lower pain scores and better functional recovery, with a higher percentage of patients regaining full mobility within six months.

## Figures and Tables

**Table 1:** Demographic and baseline characteristics of study population

**Characteristic**	**Experimental Group (3D Plates)**	**Control Group (Traditional Plates)**	**Total (n=50)**
Number of Patients (n)	25	25	50
Age (mean ± SD)	38.4±8.7	37.9±9.2	38.2±8.9
**Gender**			
- Male (%)	18 (72%)	17 (68%)	35 (70%)
- Female (%)	7 (28%)	8 (32%)	15 (30%)
**Fracture Type**			
- Angle Fractures (%)	15 (60%)	14 (56%)	29 (58%)
- Body Fractures (%)	10 (40%)	11 (44%)	21 (42%)
Right Limb Fracture (%)	13 (52%)	12 (48%)	25 (50%)
Left Limb Fracture (%)	12 (48%)	13 (52%)	25 (50%)
Mean Fracture Severity (Score)	6.2±1.3	6.3±1.4	6.25±1.35

**Table 2:** Comparison of bony union time and functional recovery

**Outcome Measure**	**Experimental Group (3D Plates)**	**Control Group (Traditional Plates)**	**p-value**
Bony Union Time (weeks)	8.5±1.2	12.3±1.5	<0.05
Functional Recovery (VAS for pain) - 1 month	3.2±1.4	5.1±2.0	<0.01
Functional Recovery (FIM Score) - 3 months	24.5±3.1	20.5 (±3.8)	<0.05
**Outcome Measure**			
Complications (%)	8%	40%	<0.05
Surgical Time (minutes)	85 (±12)	95 (±14)	N/A
Hospital Stay (days)	4.5 (±1.3)	5.2 (±1.5)	N/A
Full Weight-Bearing Time (weeks)	9.2 (±1.1)	13.4 (±1.4)	<0.05

**Table 3:** Functional recovery at different time points

**Time Point**	**Experimental Group (3D Plates)**	**Control Group (Traditional Plates)**	**p-value**
1 Month - VAS (Pain Score)	3.2±1.4	5.1±2.0	<0.01
3 Months - FIM Score	24.5±3.1	20.5±3.8	<0.05
6 Months - Full Mobility (%)	80%	62%	<0.05
